# Rational combinations of immunotherapeutics that target discrete pathways

**DOI:** 10.1186/2051-1426-1-16

**Published:** 2013-09-23

**Authors:** Stefani Spranger, Thomas Gajewski

**Affiliations:** 1 Biological Sciences Division, Pathology, The University of Chicago, 929 E. 57th Street, GCIS W-423, Chicago, IL 60637, USA; 2 Department of Pathology and Department of Medicine, Section of Hematology/Oncology, The University of Chicago, 5841 S. Maryland Ave., MC2115, Chicago, IL 60637, USA

**Keywords:** Cancer, Immunotherapy, Interferon, PD-1, PD-L1, CTLA-4, Tumor-associated antigen, Indoleamine-2,3,-dioxygenase, Denileukin diftitox, Regulatory T cell

## Abstract

An effective anti-tumor immune response requires the coordinated action of the innate and adaptive phases of the immune system. Critical processes include the activation of dendritic cells to present antigens, produce cytokines including type I interferons, and express multiple costimulatory ligands; induction of a productive T cell response within lymph nodes; migration of activated T cells to the tumor microenvironment in response to chemokines and homing receptor expression; and having effector T cells gain access to antigen-expressing tumor cells and maintain sufficient functionality to destroy them. However, tumors can become adept at escaping the immune response, developing multiple mechanisms to disrupt key processes. In general, tumors can be assigned into two different, major groups depending on whether the tumor there is an ‘inflamed’ or ‘non-inflamed’ tumor microenvironment. Improvements in our understanding of the interactions between the immune system and cancer have resulted in the development of various strategies to improve the immune-mediated control of tumors in both sub-groups. Categories of major immunotherapeutic intervention include methods to increase the frequency of tumor antigen-specific effector T cells in the circulation, strategies to block or uncouple a range of immune suppressive mechanisms within the tumor microenvironment, and tactics to induce de novo immune inflammation within the tumor microenvironment. The latter may be particularly important for eliciting immune recognition of non-inflamed tumor phenotypes. The premise put forth in this review is that synergistic therapeutic effects in vivo may be derived from combination therapies taken from distinct “bins” based on these mechanisms of action. Early data in both preclinical and some clinical studies provide support for this model. We also suggest that optimal application of these combinations may be aided by appropriate patient selection based on predictive biomarkers.

## Introduction

With a more detailed understanding of the interactions between the human immune system and cancer, and a larger armamentarium of immunotherapeutic agents in development than ever before, the field of tumor immunotherapy is growing rapidly. Progress will depend upon rational patient selection and logical development and application of these novel therapies, alone or in combination with other treatments. This review summarizes the mechanistic steps involved in the generation and regulation of anti-tumor immune responses, considers discrete categories of immunotherapies based upon type and temporal − spatial aspects of the biologic step being regulated, describes opportunities for selection of patients most likely to benefit from immunotherapy, and suggests immunotherapy combinations that may be attractive for clinical investigation based on logical subdivisions.

### The generation of spontaneous anti-tumor immune responses

Although the theory of immune surveillance remains controversial [1,2], certain pieces of experimental and observational evidence support its existence. The observation that endogenous interferon gamma (IFN-γ) and also IFN-α/β can contribute to protection against the growth of methylcholanthrene-induced fibrosarcomas implies that IFN signaling plays a key role in the immune protection against murine cancer [2-4]. Furthermore, human cancer incidence is increased in patients who are immunosuppressed or have immunodeficiencies [5-7] compared with healthy hosts. It has also been observed that melanoma and other cancers can be transmitted from organ transplant donors to recipients, once the organ recipient is immunosuppressed [8]. In light of these data, the premise remains that the immune system can contribute to control of cancer development and/or progression. As a tumor does develop, immune sensing and subsequent immune-mediated control passes through multiple physiological phases, each of which is tightly regulated.

**Figure 1:**
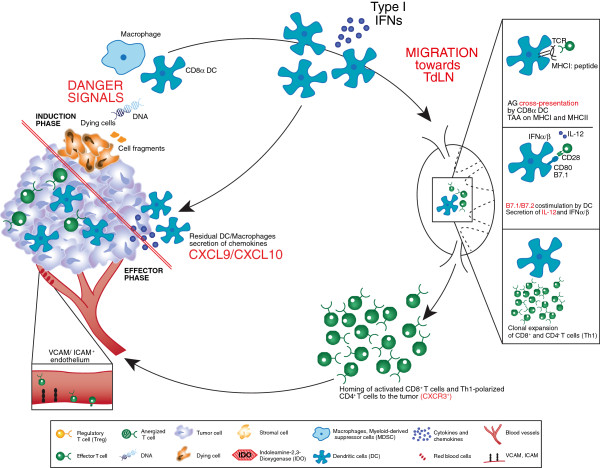
**Processes involved in an anti-tumor immune response resulting in a tumor with an “inflamed” immunophenotype.** Processes in red are those considered particularly crucial for the development of effective anti-tumor immunity. The immune response begins with the induction phase, where activated dendritic cells prime T cells, this leads to the effector phase where activated, tumor-specific T cells infiltrate the tumor microenvironment. See the Key for definitions of graphics.

The development of an anti-tumor response is a coordinated, multifaceted phenomenon comprising both the innate and adaptive phases of the immune system (Figure 1). The complex nature of this response, combined with our growing understanding of the process, offers several opportunities for clinical intervention. A brief working model of the generation of an anti-tumor immune response is summarized below.

#### Role of the innate immune system

Currently, it is hypothesized that sensors expressed by innate immune cells (e.g., dendritic cells [DC]) can detect damage-associated molecular recognition elements, likely derived from dying cancer cells that result in productive DC activation. This leads to expression of multiple chemokines that recruit additional cell types, and also upregulates expression of multiple costimulatory ligands and secreted cytokines that promote T cell activation. In the mouse, data suggest that the subset of DCs responsible for cross-presentation of antigen to T cells in a class I major histocompatibility complex (MHC)-restricted fashion is the CD8α^+^ DC subset [9,10]. Indeed, Batf3^−/−^ mice that are deficient in this lineage fail to generate a spontaneous anti-tumor T cell response [10,11]. The phenotype of the corresponding DC subset in humans has recently been elaborated, as defined by the expression of DNGR1 [12], and investigation into the involvement of this DC subset in human tumors is being evaluated. Interestingly, the activation of those DCs depends, at least in part, on the induction of IFN-α/β production in response to a growing tumor [11,13]. Type I IFN receptor^−/−^ mice, or mice deficient in the downstream signaling molecule Stat1, also fail to prime a spontaneous anti-tumor T cell response [11,13]. The innate immune sensing pathway, as well as the tumor-derived ligand(s) responsible for type I IFN production, are being elucidated and are topics of active investigation.

#### Role of the adaptive immune system

Once DCs are properly activated in response to a growing tumor, the induction of productive T cell responses against tumor-associated antigens depends on several molecular elements. Antigen cross-presentation that depends on TAP transporters and class I MHC is critical [14], and recent work has suggested that the receptor Clec9a, highly expressed by CD8α^+^ DCs, is involved with proper antigen processing [15,16]. Expression and costimulation by CD80/CD86 by host cells is required [14], as is production of interleukin (IL)-12 [17]. Mice deficient in any of these factors show poor T cell priming and defective immune-mediated tumor control.

At the effector phase of the anti-tumor T cell response, activated T cells must traffic to the tumor microenvironment. This process likely depends on the local production of specific chemokines, such as CXCL9 and CXCL10 [18]. In addition, it is thought that the vascular endothelial cells must be activated and express key homing receptors, such as ICAM-1 and VCAM-1, for T cells to transit into the tumor tissue. Buckanovich and colleagues have identified the endothelin B receptor as one regulator of this process [19]. Evidence suggests that both CD4^+^ and CD8^+^ effector cells can participate in the effector phase of the anti-tumor immune response [14,20]. Once present within the tumor site, activated T cells must maintain their functional properties (cytolytic activity, inflammatory cytokine production, and likely proliferation) and also gain access to individual antigen-expressing tumor cells. Therefore, features of the tumor microenvironment can have a major impact on whether activated T cells can effectively destroy a tumor. Based on this model, it is not difficult to imagine that immune escape by cancers might be attributed to defective T cell trafficking, suppression of T cell function, or physical limitation of access to tumor cells. However, the mechanisms of immune escape might be distinct in different patients with the same cancer and in patients with different cancer histologies.

## Review

### Strategies to increase the frequency of anti-tumor T cells

One of the longest pursued approaches to improve immune-mediated control of cancer is via strategies to increase the number of effector T cells that can potentially recognize and destroy tumor cells in vivo. These strategies involve both quantitative and qualitative considerations.

#### Vaccines

In contrast to classical prophylactic vaccines with the goal to induce an immune response before encountering the antigen, anti-tumor vaccines aim to augment immune responses with the antigen-expressing targets already present. In addition, while prophylactic vaccines largely aim to induce neutralizing antibodies, therapeutic cancer vaccines principally target induction of antigen-specific T cells. The general composition of vaccines includes a source of tumor-associated antigen (TAA) and an adjuvant component that results in activation of DCs for productive presentation. A wealth of TAAs has been molecularly defined, and this topic has been extensively reviewed [21-25]. Antigens can be incorporated into vaccines as defined proteins or peptides; tumor cell-derived preparations of protein, RNA, or crude extracts; whole tumor cells, either irradiated or engineered to secrete cytokines; or recombinant cDNAs engineered into viral or bacterial vectors. The adjuvant component can consist of oil-based formulations, defined toll-like receptor (TLR) ligands, recombinant cytokines, or the natural innate ligands associated with viral or bacterial vectors. Alternatively, to have full control over their maturation status, DCs loaded with antigen directly can be prepared and injected. Immunologic monitoring for a biologic effect of vaccines is typically performed by measuring the frequency of specific T cells in peripheral blood. The first FDA-approved therapeutic cancer vaccine is sipuleucel-T for prostate cancer, which consists of the prostatic acid phosphatase antigen fused to granulocyte-macrophage colony-stimulating factor (GM-CSF), loaded onto autologous peripheral blood mononuclear cells [26]. The GM-CSF fusion is thought to target antigen-loading onto DCs. Other vaccines in late phase development include the MAGE-3 protein-based vaccine from GlaxoSmithKline (Brentford, United Kingdom) that incorporates TLR4 and TLR9 ligands as part of the adjuvant [27,28]; and PROSTVAC® (Bavarian Nordic A/S, Kvistgaard, Denmark), which utilizes recombinant viral vectors [29]. Thus far, the clinical activity of vaccines has been modest as single agents, likely because of downstream resistance mechanisms that overpower the increased T cell frequency that is induced following immunization. Thus, combination therapies are appropriate to consider with vaccines as resistance mechanisms continue to be uncovered.

#### Adoptive T cell transfer

An alternative strategy to increase the frequency of tumor antigen-specific T cells is through adoptive T cell transfer. The general concept is to expand in vitro large numbers of tumor antigen-specific T cells, thus bypassing the early stages of endogenous T cell activation. The most successful of these approaches to date is arguably that based on tumor-infiltrating lymphocytes (TIL) developed by Rosenberg and colleagues for melanoma [30,31]. In that strategy, a tumor is resected and TIL are grown out of the tumor explant in vitro. Prior to T cell infusion, the patient is conditioned with a lymphodepleting regimen, and then is given IL-2 post infusion. Using this approach, response rates of 50% or greater have consistently been observed. However, it is important to remember that not all patients have TIL grow out or remain clinically stable at the time the expanded TIL are prepared, so the response rate based on the intent-to-treat population is likely to be lower. Alternative strategies for adoptive T cell therapy include the administration of antigen-specific T cell clones, either CD4^+^ or CD8^+^[32-34]; engineering autologous T cells to express a defined T cell receptor (TCR), either wild-type or mutated towards a higher affinity [35]; or the genetic engineering of novel receptors consisting of a chimera between an antibody molecule and TCR segments (chimeric antigen receptor) for transduction into autologous T cells [36]. Mechanistically, for solid tumors, the infused T cells still must traffic to tumor sites, penetrate the tumor microenvironment, and remain functional there. Thus, downstream resistance mechanisms may still be rate limiting in many cases. It is thought that the lymphodepleting conditioning regimen may diminish the contribution of some of these inhibitory mechanisms, as discussed further below.

#### Cytokines for T cell expansion

If a low level of endogenous T cell priming has occurred in some patients, then it is reasonable to consider that expansion of those activated T cells with T cell growth factors might raise frequencies sufficiently to gain clinical activity. The first cytokine FDA-approved for this purpose is IL-2, for the treatment of patients with metastatic melanoma and kidney cancer [37], although the mechanism of action of this agent in patients has never been firmly established. More recently explored cytokines that act, in part, by expansion of T cells include IL-7 [38], IL-21 [39], and IL-15 [40]. Interestingly, IL-7 and IL-15 have also been shown to reverse T cell anergy [41,42], so these cytokines also may theoretically restore the function of T cells rendered anergic in the tumor microenvironment (a topic discussed further below).

#### Manipulation of costimulatory pathways that function in secondary lymphoid organs

Given the critical role for costimulatory receptors in regulating T cell activation, pharmacologic manipulation of these pathways has continued to be pursued as a therapeutic approach. This includes the development of agonistic agents that ligate positive costimulatory receptors, as well as blocking agents that attenuate signaling through inhibitory receptors. While many of these pathways may be operational downstream in the tumor microenvironment, some are likely dominantly acting in secondary lymphoid structures, as that is where high expression of respective ligands is seen, usually on antigen-presenting cells. The first of these agents approved by the FDA is ipilimumab (Bristol-Myers Squibb, New York, NY, USA), a monoclonal antibody against the inhibitory receptor cytotoxic T-lymphocyte antigen-4 (CTLA-4), for metastatic melanoma [43]. Agonistic antibodies against the positive costimulatory receptors 4-1BB (CD137) [44] and OX40 [45] also have shown efficacy in preclinical models and are undergoing early phase clinical trial testing in cancer patients. It is interesting to note that these receptors are upregulated on T cells after initial TCR ligation, so the biologic activity of the above agents is likely limited to T cells already undergoing antigen recognition. There is concern with engaging costimulatory receptors constitutively expressed on resting T cells, such as CD28, as this may cause a more global T cell activation and have increased toxicity. This certainly was observed with an anti-CD28 monoclonal antibody being evaluated as a potential treatment for autoimmunity [46]. The related CD28 family member, inducible T-cell costimulator (ICOS), is inducibly expressed upon T cell activation, and preclinical data engaging ICOS via expression of ICOS-L in a vaccine preparation have shown anti-tumor effects in vivo [J.P. Allison, personal communication]. Clinical development of agonistic antibodies against ICOS should therefore receive priority. In addition to the activity of anti-CTLA-4 mAb on lowering the threshold for activation of T cells in lymphoid organs, recent data suggest that some anti-CTLA-4 mAbs also can deplete Tregs within the tumor microenvironment [47].

### Targeting immunologic barriers in the tumor microenvironment

**Figure 2:**
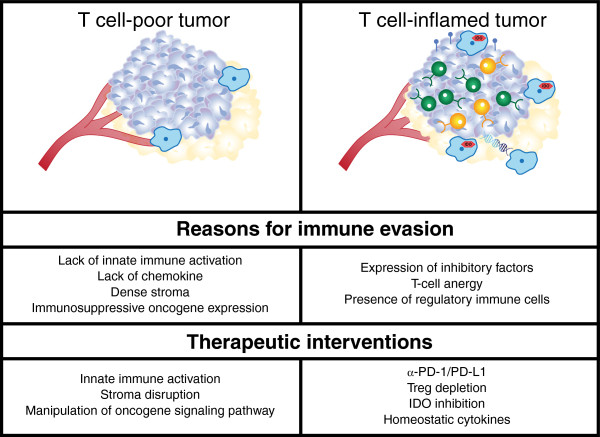
**Differences between tumors with “inflamed” and “non-inflamed” immunophenotypes and potential therapeutic interventions.** See the Figure 1 Key for definitions of graphics.

Data accumulated over several years have indicated that at least two major immunophenotypes of metastatic cancer likely exist. One major phenotype is characterized by the presence of activated CD8^+^ T cells, expression of chemokines, and also indicators of innate immune activation such as a type I IFN transcriptional signature (Figure 2). The other phenotype looks non-inflamed and shows evidence for higher levels of angiogenesis, macrophage-lineage cells, and fibroblasts in addition to cancer cells. It is likely that the major barriers to immune-mediated tumor destruction differ between these two subsets, and early clinical data support this working model.

#### Targeting immune inhibitory pathways expressed in T cell-inflamed tumors

**Figure 3:**
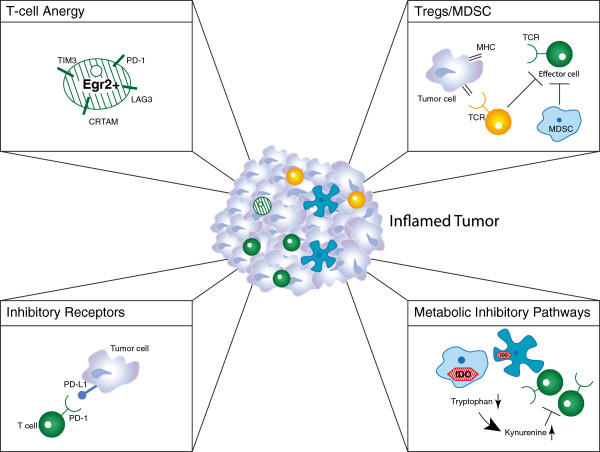
**Dominant inhibitory mechanisms in the tumor microenvironment that suppress anti-tumor immunity.** See the Figure 1 Key for definitions of graphics.

It may seem paradoxical that a subset of tumors can be replete with activated CD8^+^ T cells yet the tumor is nonetheless growing progressively. In HLA-A2^+^ melanoma patients, analysis of small series of samples has indicated that tumor antigen-specific T cells are among those present, yet these T cells appear to be dysfunctional [48-50]. Thus, despite the presence of chemokines that have promoted recruitment of activated T cells, it appears that dominant inhibitory mechanisms have resulted in a loss of function in these cases (Figure 3). Importantly, strategies aiming to block or reverse these inhibitory mechanisms are being pursued clinically. The immune suppressive mechanisms that are best characterized and are farthest along in terms of targeting in the clinic are the inhibitory receptor programmed death 1 (PD-1), which is engaged by the ligand programmed death-ligand 1 (PD-L1, also called B7-H1) expressed by tumor cells; tryptophan catabolism by the enzyme indoleamine-2,3-dioxygenase (IDO); extrinsic suppression by CD4^+^CD25^+^FoxP3^+^ regulatory T cells (Tregs); and T cell-intrinsic anergy or exhaustion, that is best characterized to result from TCR ligation in the absence of engagement of costimulatory receptors such as CD28 [51,52]. Recent data in melanoma have revealed that the presence of these immune suppressive mechanisms is highest in tumors that contain infiltrating T cells, and that activated CD8^+^ T cells are major mediators of their recruitment. The upregulation of PD-L1 and IDO appear to be driven by T cell-derived IFN-γ, and the accumulation of Tregs appears to be driven by T cell-derived chemokines [53,54]. Thus, these major mechanisms of immune suppression in the tumor microenvironment are likely immune-intrinsic rather than directly tumor-induced.

#### Inhibitory receptors: PD-1/PD-L1 interactions

PD-1 is an inhibitory receptor inducibly upregulated on activated T cells [55]. The major ligand for PD-1, PD-L1, can be expressed directly on tumor cells. Thus, this receptor/ligand interaction is active within the tumor microenvironment. Preclinical models have demonstrated that blockade of PD-1 or PD-L1, or the use of PD-1-deficient T cells, can result in profound immune-mediated tumor control in many experimental systems [56-59]. Multiple human cancer types have been demonstrated to express PD-L1 in the tumor microenvironment [60]. Clinically, monoclonal antibodies targeting PD-1 or PD-L1 have already shown major clinical activity in phase I/II clinical trials, with response rates around 30% in patients with melanoma, kidney cancer, and non-small cell lung cancer [55,61-64]. These agents also have encouraging safety profiles, although treatment-associated adverse events have included instances of pulmonary toxicity. Phase III studies with nivolumab (anti-PD-1; Bristol-Myers Squibb, New York, NY, USA) monotherapy are ongoing in melanoma, squamous and non-squamous non-small cell lung cancer, and kidney cancer. Phase II studies in other malignancies and phase I combination studies with various anti-PD-1 monoclonal antibodies have all been initiated.

In addition to PD-L1, at least two other inhibitory ligands have been reported to be expressed on tumor cells, namely, B7-H3 and B7-H4. Expression of these ligands correlates with poorer outcome or more advanced disease in some tumor types and preclinical data have supported efficacy with blocking antibodies in vivo [65-67]. Clinical development of antibodies specific for human counterparts is warranted.

#### Metabolic dysregulation: IDO

T cell-infiltrated melanomas and other tumor types also appear to show increased expression of the immunosuppressive enzyme IDO. In most tumor types examined, IDO expression has correlated with unfavorable patient prognosis and is associated with advanced stage and tumor metastasis [68]. In normal physiology, IDO metabolizes tryptophan and limits T- and NK-cell activation in local tissue microenvironments, such as the placenta [69,70]. IDO expression in preclinical models prevents tumor rejection, and blockade of IDO activity can be immune-potentiating in vivo [71,72]. Two small-molecule IDO inhibitors are in clinical development, INCB024360 (Incyte Corporation, Wilmington, DE, USA) [73-75] and 1-methyl-DL-tryptophan [76-78]. Phase I clinical trial data presented at the American Society of Clinical Oncology 2012 meeting showed that, in patients treated with INCB024360, biologically active doses were achieved causing a reversal of the tryptophan to kynurenine ratio [76]. Phase II single agent and phase I combination studies have been initiated with these agents.

A second amino acid-catabolizing enzyme expressed in the tumor microenvironment is arginase, which catabolizes arginine [79]. Preclinical data indicate that arginine metabolism is one way in which T cell dysfunction can occur within the tumor microenvironment, and conditional knockout mice in which arginase is eliminated in myeloid cells show improved anti-tumor T cell responses [79]. However, targeting this enzyme pharmacologically in the clinic might prove challenging due to baseline expression and function in normal tissues.

#### Suppressive cell populations: Tregs and MDSCs

In normal physiology, Tregs thwart the development of autoimmunity and curb bystander tissue destruction by limiting ongoing immune responses and maintaining tolerance to self-antigens [80,81]. In cancer patients, circulating Treg numbers are reported to be increased compared with normal controls [82-84]. In addition, high numbers of infiltrating Tregs have been observed in the tumor microenvironment in a subset of patients with various cancers. As mentioned above, recent data in melanoma indicate that higher Treg numbers are observed in tumors that have greater infiltration with CD8^+^ T cells [53]. There are two major populations of Tregs, those that develop naturally in the thymus and those that are induced in the periphery under the influence of TGF-β [85]. Our own observations in the B16 melanoma model have revealed that natural Tregs are the major subset recruited into the tumor microenvironment rather than conversion of FoxP3-negative CD4^+^ T cells [53]. In ovarian cancer, a higher CD8:Treg ratio has been correlated with improved overall outcome [86]. In preclinical models, depletion of Tregs using anti-CD25 monoclonal antibody or by ex vivo anti-CD25 bead depletion has been shown to improve immune-mediated tumor control in vivo [87-89]. Based on these observations, strategies to deplete Tregs in cancer patients are being pursued using a variety of approaches. To date, all of these approaches are focusing on targeting CD25. The IL-2/diphtheria toxin fusion protein, denileukin diftitox (Ontak®, Eisai Inc., Woodcliff Lake, NJ, USA), has been reported to diminish Treg numbers in the circulation in some studies [90-92], although this has not been observed in all trials [93]. Various doses and schedules are being pursued, and clinical regressions have been reported in melanoma [94]. The anti-CD25 monoclonal antibody daclizumab (F. Hoffmann-La Roche Ltd., Basel, Switzerland) also has been given to advanced cancer patients and has been shown to decrease Treg numbers in the peripheral blood for a prolonged period of time [95]. A third strategy being evaluated involves immune-bead depletion of CD25^+^ cells from T cell products prior to adoptive transfer into patients.

In addition to Tregs, a second extrinsically suppressive cellular population that can be active within the tumor microenvironment is represented by myeloid-derived suppressor cells (MDSCs). These cells appear to consist of immature myeloid populations that both support tumor growth and inhibit T cell activation via a number of mechanisms. This includes the expression and functional activity of arginase [79], and the nitrosylation of surface proteins on infiltrating T cells, including the TCR [96]. Therapeutic approaches to diminish MDSC number or function are challenging due to difficulties identifying specific pharmacologic targets, but several interventions are being tested in patients with cancer [97].

#### T cell-intrinsic anergy

In addition to the above-listed extrinsic mechanisms of inhibition of T cell function, recent evidence supports a role for T cell-intrinsic anergy as a contributory mechanism of immune evasion in the tumor microenvironment. Classical anergy is a dysfunctional state that results from TCR ligation in the absence of costimulatory receptor engagement [98]. Preclinical and clinical data analyzing TIL in melanoma and other models indicate that purified antigen-specific T cells remain dysfunctional early after removal from the immune suppressive influence of the tumor microenvironment [48-50]. Early data suggested that introduction of the CD28 ligand B7-1 (CD80) into tumor cells could result in immune-mediated rejection in vivo [99-101]. Unlike the extrinsic mechanisms of suppression described above, it has been more difficult to consider targeting anergy therapeutically because of lack of molecular targets suitable for manipulation. However, recent molecular characterization of the anergic state has provided new insights that are relevant for therapeutic intervention. In vitro, anergic T cells can recover function following proliferation in response to homeostatic cytokines (IL-7, IL-15, and perhaps IL-21) [41]. In vivo, endogenous IL-7 and IL-15 are liberated under conditions of lymphopenia [102,103]. Adoptive transfer of anergic T cells into lymphopenic recipients can reverse T cell anergy and support tumor rejection [104]. The ability of a lymphopenic environment to maintain T cell function may explain, in part, the success of TIL-based adoptive transfer regimens that include lymphopenic conditioning [30,31,105].

Recent work has identified the transcription factor early growth response gene 2 (EGR2) as a regulator of the anergic state [106]. EGR2 is upregulated in anergic cells, and in part functions by driving expression of diacylglycerol kinases, which inhibit TCR-induced Ras pathway activation [107,108]. EGR2-dependent gene expression profiling and ChIP-SEQ analysis have revealed additional EGR2 target genes that are functionally important [109]. Some of these encode surface proteins, including LAG-3 and 4-1BB. LAG-3 has been defined as another inhibitory receptor expressed on activated T cells [110,111], and 4-1BB is a costimulatory receptor [44,112]. Dysfunctional T cells in the tumor context also have been shown to express Tim-3, another inhibitory receptor [113]. Preclinical data show that blockade of LAG-3 or Tim-3, or ligation of 4-1BB, can potently augment immune-mediated tumor rejection in vivo [114-116]. Taken together, these results suggest that manipulation of these receptors might operate, at least in part, to restore function of anergic anti-tumor T cells.

#### Overcoming barriers in non-inflamed tumors

Tumors that fail to generate a spontaneous anti-tumor T cell response and lack a T cell infiltrate may represent a special case from the immunotherapy perspective and could require additional interventions to enable immune recognition. The underlying mechanisms responsible for lack of a T cell-inflamed tumor microenvironment are not fully understood, but this phenotype correlates with absence of a type I IFN signature and poor chemokine production, suggesting defective innate immune activation and a deficient ability to recruit activated T cells [18,117].

Administration of IFN-alpha had been approved by the FDA for the treatment of various cancers, including melanoma where it is used in the adjuvant setting [118,119]. However, systemic administration might not maximize the therapeutic effect as it fails to provide “directionality” to the inflammatory response. Preclinical data from our own laboratory have indicated that intratumoral administration can be more effective (unpublished data). Published preclinical studies support the notion not only that type I IFNs can help promote innate immune activation in the tumor microenvironment [120], but in addition that local application of TLR ligands [121], expression of the tumor necrosis factor superfamily member LIGHT [122,123], and injection of oncolytic viruses [124] also may have utility in this regard. Clinical studies applying these approaches are ongoing or under development but further research into the underlying mechanisms governing the absence of a spontaneous anti-tumor T cell response in a major subset of cancers is warranted to help guide the development of these therapies with greater precision. The first oncolytic virus tested in a phase III study in melanoma was recently reported to meet the primary endpoint based on clinical response [125]. One challenge facing attempts to modify inflammation in the tumor microenvironment selectively in vivo is to devise strategies for systemic administration of agents that preferentially target tumor sites. One conceptual approach would be the use of tumor-targeting monoclonal antibodies carrying immunoregulatory molecules as a payload. Another barrier for development of these agents is the lack of an ideal mouse model for preclinical development. All transplantable tumor models appear to induce a meaningful degree of inflammation, so the development of a system for non-inflamed tumors may depend on design of a genetic model of suitable oncogene combinations. Ultimately, interventions aimed at initiating inflammation in the tumor site will likely benefit from combinations with therapeutic approaches increasing the T cell frequency and blocking negative regulatory pathways, as discussed below.

### Logical immunotherapy combinations

**Figure 4:**
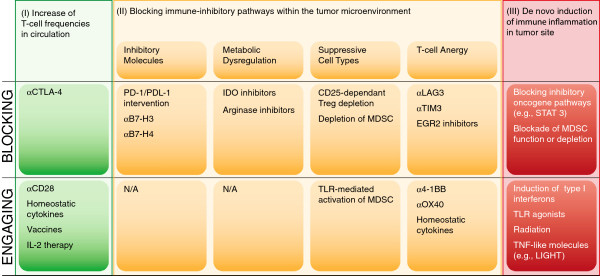
Categories of potential immunotherapeutic interventions for cancer and opportunities for combinations.

Based on the above structural overview, a model for prioritizing combination therapy testing based on distinct categories of regulatory checkpoint emerges. An overview of these considerations is depicted in Figure 4. One can consider two broad categories of patients—those with a T cell-inflamed tumor microenvironment and a spontaneous anti-tumor T cell response, and those with a non-inflamed tumor microenvironment and a minimal spontaneous anti-tumor T cell response. In addition, there are three major “bins” of interventions—strategies to increase systemically the frequency of anti-tumor T cells (I), strategies to overcome distinct immune suppressive pathways within the tumor microenvironment (II) and strategies to trigger innate immune activation and inflammation in tumor sites (III). The second category can be further subdivided into approaches to block inhibitory receptor engagement (e.g., PD-L1/PD-1 interactions), deplete Tregs, inhibit metabolic enzymes such as IDO, or reverse/prevent T cell anergy. Because each of these processes is largely regulated independently, one might expect true synergy when they are manipulated in a combinatorial fashion. A corollary is that combinations of interventions that influence the same process or pathway might not be synergistic, although they could conceivably be additive.

Multiple examples of successful immunotherapy combinations have been evaluated in preclinical models and the results are in keeping with the logic of the framework described above. For example, Treg depletion plus homeostatic proliferation in a lymphopenic recipient (to counter T cell anergy) can have potent activity in some models [14,87]. This can become even more efficacious with adoptive T cell transfer as a strategy to increase T cell frequencies [126]. Treg depletion also can be synergistic with some vaccines [89,95,127]. Combinatorial blockade with anti-CTLA-4 and anti-PD-1 monoclonal antibodies can be potently synergistic in some tumor models [128]. Anti-4-1BB monoclonal antibodies (which could act in the periphery to increase T cell frequencies or in the tumor microenvironment to restore function of anergic cells) combined with anti-PD-L1 also appear synergistic [59,129], as is combined elimination of LAG-3 and PD-1 function [130] or anti-Tim-3 plus anti-PD-L1 [131]. Preliminary data from our laboratory have indicated that IDO inhibition combined with either anti-CTLA-4 or anti-PD-L1 monoclonal antibodies can also result in potent immune-mediated tumor control in vivo [authors’ unpublished observations]. However, preliminary experiments with anti-LAG-3 and anti-4-1BB (which both may manipulate anergic T cells) have not shown synergistic effects.

Based on these and other similar preclinical data, several logical combination immunotherapies are already being evaluated in clinical trials. Several therapeutic cancer vaccines are being tested in combination with Treg depletion, using either denileukin diftitox or anti-CD25 monoclonal antibody [75,132-134]. The Cancer Immunotherapy Trials Network is planning to investigate the prostate cancer vaccine sipuleucel-T along with the homeostatic cytokine IL-7 [http://citninfo.org/citn-science/clinical-studies.html]. The anti-CTLA-4 monoclonal antibody ipilimumab is being tested in combination with an IDO inhibitor [75] and also with nivolumab in a phase III trial in patients with previously untreated metastatic melanoma [135]. Phase I/II data of anti-CTLA-4 + anti-PD-1 showed a clinical response rate of over 50%, with more rapid and deep clinical responses than what had been observed historically with either agent alone [136]. TIL-based adoptive T cell therapy has already been shown to be most potent when combined with lymphopenic conditioning of the patient, which is thought to reduce Treg numbers and support homeostatic proliferation of transferred T cells [30,31,105].

The clinical application of multiple immunotherapies in combination will require careful consideration of several factors, including the timing of agent administration (concurrent vs sequential, as previously evaluated), the potential for overlapping/additive toxicities of the individual agents, and particularly the development of synergistic toxicities, including potential sequelae of immune system overstimulation. However, with appropriate adverse-event management, treatments targeting multiple, discrete branches of tumor-associated immunity may have the potential to improve patient outcomes dramatically.

### Biomarkers

The successful application of combination immunotherapies in the clinic may ultimately benefit from appropriate patient selection based upon predictive biomarkers. Based on available data, a leading biomarker for response to current immunotherapies is the presence of an “inflammatory” gene expression signature that suggests an ongoing, smoldering immune response against the tumor. The predictive significance of these signatures has been preliminarily confirmed in several small studies [137-141]. A similar correlation has been reported with nivolumab, in which clinical responses appear associated with expression of PD-L1 in the tumor microenvironment along with a CD8^+^ T cell infiltrate [63,75,135]. Combination immunotherapies that manipulate the endogenous immune response or involve strategies to increase the frequency of anti-tumor T cells may all rely on the intrinsic ability of the metastatic tumor sites to recruit effector T cells into the tumor microenvironment. More specific markers could be envisioned as predictive for benefit to anti-LAG-3, anti-Tim-3, or anti-4-1BB, for example, based on the presence of T cells in the tumor microenvironment that are dysfunctional, yet show surface expression of these receptors ex vivo.

In contrast, patients with tumors that are “non-inflamed” may respond poorly to most of these immunotherapeutic interventions because there is no spontaneous endogenous immune response to be manipulated, and/or because activated T cells cannot traffic into the tumor microenvironment. Therefore, such patients ultimately might require new strategies to induce appropriate innate immune activation and chemokine production in the tumor microenvironment. One can envision combining these strategies with approaches to increase the frequency of anti-tumor T cells and/or block negative regulatory pathways in the tumor microenvironment.

## Conclusions

Mechanisms of tumor immune escape are multiple and can compensate for one another, and preclinical models suggest synergy when two distinct mechanisms are manipulated in concert. It is anticipated that logical doublet combinations in the clinic will impart a meaningful impact on patient outcomes. Different cancer types beyond melanoma may have specific dominant mechanisms of suppression (e.g., B7-H3, B7-H4), and therefore could benefit from unique, tailored immunotherapy combinations. Finally, one of the biggest challenges might be to promote T cell-based inflammation in “non-inflamed” tumors in order to expand the subset of patients in whom currently active immunotherapies appear effective. With a broader arsenal of immunotherapeutic agents and a deeper understanding of tumor-host interactions, clinical tumor immunotherapy is poised to advance significantly. Careful consideration of appropriate patient candidates based on biomarker development and a logical, coordinated application of immunotherapy combinations should accelerate advancement of the field.

## Abbreviations

CTLA-4: Cytotoxic T-lymphocyte antigen 4; DC: Dendritic cell; EGR2: Early growth response gene 2; GM-CSF: Granulocyte-macrophage colony-stimulating factor; ICOS: Inducible T-cell costimulator; IDO: Indoleamine-2,3-dioxygenase; IFN: Interferon; IL: Interleukin; MDSC: Myeloid-derived suppressor cells; MHC: Major histocompatibility complex; PD-1: Programmed death-1; PD-L1: Programmed death – ligand 1; TAA: Tumor-associated antigen; TCR: T cell receptor; TIL: Tumor-infiltrating lymphocytes; TLR: Toll-like receptor; Treg: Regulatory T cell.

## Competing interests

TG has served in an advisory role for GSK-Bio, Roche-Genentech, BMS, Merck, Abbvie, and Jounce Therapeutics. SS declares no competing interests.

## Authors’ contributions

TG and SS both wrote and revised the manuscript, with editorial assistance from Cailin Moira Wilke, PhD, at StemScientific. Both authors read and approved the final manuscript.
